# Morphological characters of the immature stages of Tychius (Apeltarius) amplicollis Aubé, 1850 (Coleoptera, Curculionidae, Curculioninae, Tychiini), supported by molecular and biological data, suggest that *Apeltarius* Desbrochers des Loges, 1873 is a new synonym of *Tychius* Germar, 1817

**DOI:** 10.3897/zookeys.1272.183226

**Published:** 2026-03-10

**Authors:** Roberto Caldara, Jiří Skuhrovec, Roberto Casalini, Gael J. Kergoat, Roland Godon, Julien Haran, Rafał Gosik

**Affiliations:** 1 World Biodiversity Association Onlus, c/o Museo Civico di Storia Naturale, 37129 Verona, Italy Maria Curie-Skłodowska University Lublin Poland; 2 Group Function of Invertebrate and Plant Biodiversity in Agro-Ecosystems, Czech Agrifood Research Center, Prague 6–Ruzyně, Czech Republic Museo Civico di Zoologia Roma Italy; 3 Museo Civico di Zoologia, Via Ulisse Aldrovandi, 18, I–00197 Roma, Italy Group Function of Invertebrate and Plant Biodiversity in Agro-Ecosystems, Czech Agrifood Research Center Prague Czech Republic; 4 CBGP, INRAE, IRD, CIRAD, Institut Agro, Université de Montpellier, Montpellier, France CBGP, INRAE, IRD, CIRAD, Institut Agro, Université de Montpellier Montpellier France; 5 DIADE, IRD, CIR, AD, Université de Montpellier, Montpellier, France DIADE, IRD, CIR, AD, Université de Montpellier Montpellier France; 6 Zoological Museum, Faculty of Biology and Biotechnology, Maria Curie-Skłodowska University, Akademicka 19, 20–033 Lublin, Poland World Biodiversity Association Onlus, c/o Museo Civico di Storia Naturale Verona Italy

**Keywords:** Host plant, mature larva, mitochondrial COI, life habits, pupa, taxonomy, weevil

## Abstract

*Tychius* is a speciose and widespread weevil genus, whose phylogenetic relationships are largely unknown. Here we assess the validity of the subgenus *Apeltarius* using an integrative approach combining larval morphology and molecular data. The mature larva and pupa of Tychius (Apeltarius) amplicollis Aubé, 1850 are described and illustrated in detail for the first time. Comparisons with known immature stages of several species of *Tychius* s. str. reveal that the larva and pupa of *T. amplicollis* exhibit all diagnostic features characteristic of the genus *Tychius*, showing closest affinity to T. (Tychius) quinquepunctatus (Linnaeus, 1758). Phylogenetic analysis of a fragment of the mitochondrial cytochrome c oxidase subunit I gene confirms their close relationship and supports the inclusion of *T. amplicollis* in the genus *Tychius*. Newly obtained biological data show that the life history and host-plant associations of *T. amplicollis* match those of other *Tychius* species, with Fabeae (Fabaceae) as hosts. Based on this evidence, we propose that *Apeltarius* be treated as a junior subjective synonym (**syn. nov**.) of *Tychius*. Because there are no other subgenera in the genus *Tychius* we propose the following combinations: *Tychius amplicollis* Aubé, 1850; *Tychius multilineatus* (Desbrochers des Loges, 1873); *Tychius quinquelineatus* Tournier, 1874; *Tychius strigulatus* (Desbrochers des Loges, 1875).

## Introduction

The weevil tribe Tychiini is a group with a convoluted taxonomic history and currently consists of six genera placed in three subtribes (Demimaeina, Lignyodina, and Tychiina) (Caldara et al. 2014). A recent phylogenomic study (Haran et al. 2023) showed that the tribe, as currently defined, is not monophyletic due to the unrelatedness of sampled representatives of the subtribes Lignyodina and Tychiina. The subtribe Tychiina consists of two genera forming a single clade: *Tychius* Germar, 1817 (ca 300 species) and *Sibinia* Germar, 1817 (ca 240 species: Caldara et al. 2014; Haran et al. 2023). The genus *Apeltarius* Desbrochers des Loges, 1873 (Curculionidae, Curculioninae, Tychiini; type species *Apeltarius multilineatus* Desbrochers des Loges, 1873) was downgraded to a subgenus of *Tychius* Germar, 1817 by Caldara (1978) on the basis of morphological similarities between adults of the two taxa. Afterwards, the other species of *Tychius* (about 300) were revised and considered as belonging to the nominotypical subgenus, which was divided into many different species groups.

Most *Tychius* species are distributed in the Palearctic region (ca 240 species; Alonso-Zarazaga et al. 2023), with the remainder found in the Afrotropical region (45 species, mainly in South Africa) and only a few in the Nearctic and Oriental regions. The Palearctic species were arranged into 22 species groups (Caldara 1990). The North American species possibly belong to four of the Palearctic groups (Clark 1971; Clark et al. 1978), whereas the Afrotropical species fall into six groups, four apparently endemic but the other two including also several Palearctic species (Caldara 1989a, 1996, 2013; Caldara et al. 2008). In the Oriental region three groups are represented, only one of them being endemic, the *T. eremita* Caldara group (Caldara 1989b).

In this context, the validity and placement of the subgenus *Apeltarius* remains to be investigated. This subgenus is currently composed of only four circum-Mediterranean species. Aside from the type species, these include *T. amplicollis* Aubé, 1850, *T. quinquelineatus* Tournier, 1874 and *T. strigulatus* (Desbrochers des Loges, 1875), which have been treated for a long time as belonging to distinct genera (Klima 1934). They were included in a unique assemblage of species with unknown biology by Caldara (1978) on the basis of unusual morphological characters of the adults, such as the shape of the rostrum, which is perfectly cylindrical from the base to the apex and in lateral view slightly concave at its base (as in some Lignyodina), the distinctly short shape of the pronotum and elytra, the shape of the tibiae, the external margin of which protrudes externally apically, the lack of wings and the fused elytra. On the other hand, Caldara (1978) also emphasized the numerous characters in common between *Apeltarius* and the type species of *Tychius* – *T. quinquepunctatus* (Linnaeus, 1758) – such as toothed mandibles, pronotum constricted at its apex, and the very similar male and female genitalia. All four species of the subgenus *Apeltarius* are rare and reported from only a few localities.

Recent field investigations conducted in central Italy revealed that *Vicia macrocarpa* (Moris) Bertol. – often treated as Vicia sativa subsp. macrocarpa (Moris) Arcang. – serves as host for T. (Apeltarius) amplicollis (Casalini et al. 2018), which is the taxon morphologically more closely related to the type species of *Apeltarius* (Caldara 1978). In addition to the adults, larvae and pupae were also collected, and thus immature stages representative of the subgenus *Apeltarius* became available for study. *Vicia macrocarpa* belongs to the family Fabaceae, consistent with the repertoire of known hosts documented for other *Tychius* species (Caldara et al. 2014).

Based on this material, the present study aims to evaluate the systematic placement of the subgenus *Apeltarius* within the tribe Tychiini. We conducted an integrative approach comprising: (1) a detailed description of the immature stages of *T. amplicollis*, with a comparison of their characters with those of other available *Tychius* species, and (2) a molecular phylogenetic analysis of the genus *Tychius* based on the 5' fragment of the mitochondrial cytochrome *c* oxidase subunit I (COI) gene corresponding to the standard barcode region. The results include an updated key for immature stages of *Tychius* as well as the relevant nomenclatural acts.

## Materials and methods

### Insect collection

The adults were collected by beating *Vicia macrocarpa* plants in a pasture, while the mature larvae were obtained in the laboratory from the pods of the same plant, which were placed in wide-mouth sterile transparent plastic containers covered with netting. Some of the larvae were preserved in tubes containing 95% ethyl alcohol, while the remaining larvae were transferred to similar containers filled with a substrate of sand and peat moss, into which they tunnelled and completed their development to pupae and adults. After a few days, some pupal cells were opened to determine the exact timing of pupation and subsequent adult emergence.

### Morphological descriptions

Some larval and pupal material was preserved in Pampel fixation liquid (Gosik and Skuhrovec 2011) and used for the morphological descriptions. To prepare the slides, we followed May (1977): a larva was decapitated, and the head was cleared in a 10% potassium hydroxide (KOH) solution and then rinsed in distilled water. After clearing, the mouthparts were separated from the head capsule, and the head capsule and all mouthparts were mounted on permanent microscope slides in Euparal. All other body parts were mounted on temporary microscope slides in 10% glycerine. The observations and measurements were conducted using a light microscope with calibrated oculars (Olympus BX 40 and Nikon Eclipse 80i). The following characters were measured for each larva: head width, body length (larvae fixed in a C-shape was measured in segments), and body width in the widest place (i.e. metathorax or abdominal segments I–IV). For the pupa, the length and width at the widest place were measured. The lengths of all setae are visible on figures. Images of details of immature morphology were taken with a HIROX digital microscope (RH-2000). Drawings were made with a drawing tablet (Intuos Pro S, Wacom, Saitama Prefecture, Japan), and the digital images subsequently processed with Adobe Photoshop, Corel Photo-Paint 11 and/or GIMP 2. The numbers of setae of bilateral structures are given for one side. We used the terms and abbreviations for the setae of the mature larvae and pupae according to Scherf (1964), May (1977, 1994), and Marvaldi (1999, 2003).

### DNA extraction, amplification, and sequencing

For this study, two specimens were sequenced for a fragment of COI corresponding to the standard 658 bp DNA barcode region (Hebert et al. 2003). DNA was extracted from a leg, using a DNeasy Blood & Tissue Kit (QIAGEN, Hilden, Germany). PCR amplification was carried out using a mix of primers for amplification of COI (Germain et al. 2013). PCR reactions were carried out in a Mastercycler Nexus (Eppendorf, Hamburg, Germany) in a final volume of 25 μl: 2 μl of genomic DNA, 17.875 μl of ultra pure water, 2.5 μl of 10×PCR buffer (final concentration = 1×), 0.5 μl of 25 mM MgCl2 (0.5 mM), 0.5 μl of each 10 μM primer cocktail (0.2 μM), 1 μl of each 2.5 mM dNTP (0.1 mM), 0.125 μl of 5 units Taq DNA Polymerase (Dream Taq, Thermo Scientific) (0.625 unit). PCR conditions for COI were: 94 °C for, five cycles of 94 °C for 30 s, 45 °C for 40 s and 72 °C for 60 s, followed by 35 cycles of 94 °C for 30 s, 51 °C for 40 s and 72 °C for 60 s, with a final extension at 72 °C for 10 min. PCR products were visualized on a 1.5% agarose gel. Unpurified PCR products were sent to Eurofins Genomics (http://www.eurofinsgenomics.eu) for sequencing using M13F (21) 5'–TGTAAAACGACGGCCAGT–3') and M13R (27) 5'–CAGGAAACAGCTATGAC–3' primers (Ivanova et al. 2007), which correspond to the “tails” added to the PCR primers. Both strands for each overlapping fragment were assembled using Geneious v. 11.1.5. All the sequences generated in this study were deposited in GenBank (see Suppl. material 1 for the accession numbers). Voucher specimens were mounted, dried and deposited at CBGP, Montpellier, France, in the CIRAD collection (https://doi.org/10.15454/D6XAKL).

### Sequence collection and alignment

COI sequences of species in the genera *Tychius* Germar, *Sibinia* Germar, and *Notolomus* LeConte, 1876 were retrieved from GenBank (Sayers et al. 2025) and BOLD (Ratnasingham et al. 2024) in September 2025. The genus *Notolomus* was used as an outgroup, following the results of the phylogenomic study of Haran et al. (2023). Sequences were aligned using MAFFT v. 7.520 (Katoh and Standley 2013) with the following parameters: mafft –localpair –reorder –maxiterate 1000–leavegappyregion –adjustdirectionaccurately –out output_file input_file. The resulting alignment was cleaned using HmmCleaner with the command HMMcleanNuc.pl –del–char – file.fasta 9, and all positions containing more than 50% missing data were removed. Two datasets were analysed. The first included all specimen sequences available for the genus *Tychius* to assess the monophyly of each species (see Suppl. materials 1–5). Based on this, a second dataset was assembled, including only one specimen per species, chosen among those having the most complete barcode sequence. The list of specimens and their corresponding accession numbers used for species-level phylogeny is provided in the Supplementary data.

### Phylogenetic reconstruction

Phylogenetic analyses were conducted using maximum likelihood with IQ–TREE v. 2.2.6 (Minh et al. 2013, 2020). The sequences were divided into three partitions (one per nucleotide position), and substitution models were determined using ModelFinder –m MFP+MERGE (Kalyaanamoorthy et al. 2017). The support of each branch was estimated using two metrics: Shimodaira–Hasegawa-like aLRT (SH–aLRT) (Guindon et al. 2010) with the –alrt 1000 option and ultrafast bootstraps (UFBOOT) (Minh et al. 2020) with the –B 1000 option.

## Results

### Tychius amplicollis

Taxon classificationAnimaliaColeopteraCurculionidae

Aubé, 1850

C60732BC-9837-5100-9B78-7553FF8C111E

#### Material examined.

Mature larvae: 10 exx., pupae: 4 ♂♂ and 4 ♀♀, adults: 30 exx. from: Italy, Latium (Rome province) road Civitavecchia–Allumiere, via Terme di Traiano 329 m, 42°6.855'N, 11°51.688'E, on 16.V.2018, leg. R. Casalini & E. Colonnelli; same locality 02.VI.2018, leg. R. Casalini & E. Colonnelli; same locality 16.V.2020, leg. R. Casalini; same locality 20.V.2024, leg. R. Casalini. On *Vicia macrocarpa*. All specimens were identified by association with adults collected on the same plant.

#### Description of mature larva.

***Measurements*** (in mm): body length: 4.70–5.80 (mean 5.20); body width: 1.7–2.2 (mean 2.0) (at the level of metathorax); head width: 0.975–1.750 (mean 1.040).

**Figure 1.**
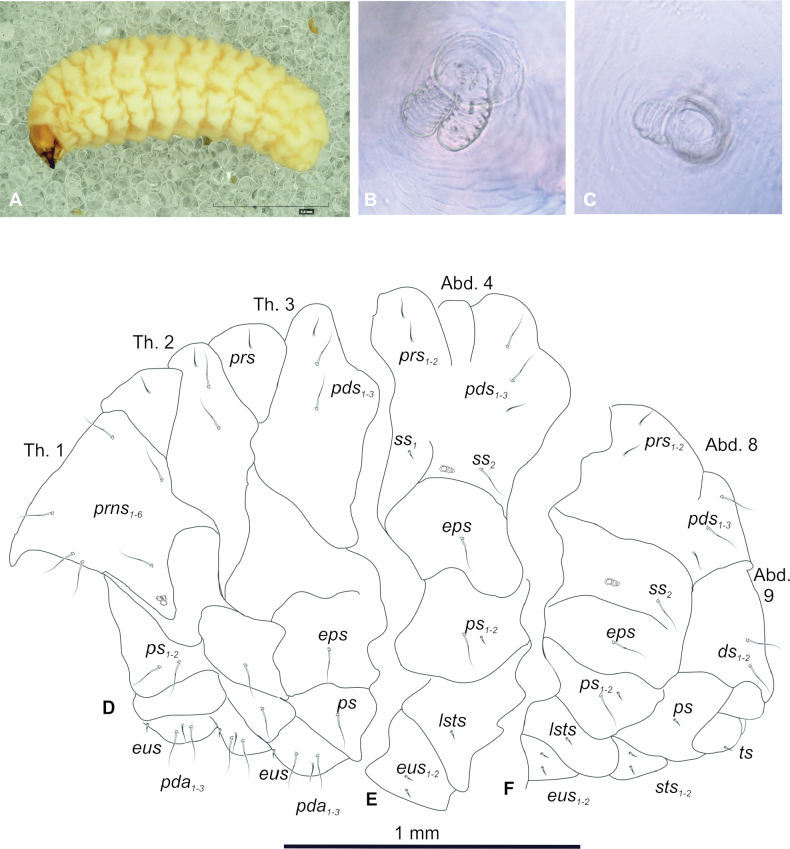
*Tychius amplicollis* mature larva. **A**. Habitus, lateral view; **B**. Thoracic spiracle; **C**. Abdominal spiracle; **D–F**. Chaetotaxy (*ds* – dorsal s., *eus* – eusternal s., *eps* – epipleural s., *lsts* – laterosternal s., *pda* – pedal s., *pds* – postdorsal s., *prns* – pronotal s., *prs* – prodorsal s., *ps* – pleural s., *ss* – spiracular s., *sts* – sternal s., *ts* – terminal s., Th.1–3 – number of thoracic segments, Abd.1–10 – number of abdominal seg.).

***Colouration***. Live larva yellow, with dark yellow head capsule; pronotal shield slightly more sclerotised on the anteromedial part (light brown) (Fig. 1A).

***General***. Body moderately stout, slightly curved, rounded in cross section. Prothorax prominent; pronotum weakly separated. Mesothorax slightly narrower than metathorax, together wider than prothorax. Meso- and metathorax each divided dorsally into two lobes (prodorsal lobe slightly wider than postdorsal). Pedal lobes of thoracic segments weakly isolated. Abdominal segment I as large as metathorax. Abd. segments II–V of similar size, wider than abdominal segment I; segments VI and VII wide; segment IX much smaller than previous one; segment X reduced, divided into three lobes (dorsal the biggest, two lateral slightly smaller, almost of equal size). Abdominal segments I–VII divided into three dorsal lobes of unequal size: prodorsal and postdorsal folds prominent, of similar size, middle fold narrow); abd. segment VIII dorsally partially divided, both folds similar in size; segment IX dorsally undivided. Epipleural and pleural lobes of abd. segments I–VIII conical, well isolated. Abd. segment IX reduced, divided into lateral, pleural and sternal lobes. Anus terminal (Fig. 1A). Thoracic spiracle bicameral (Fig. 1B) laterally placed close to border with mesothorax, abdominal spiracles unicameral (Fig. 1C) medio-laterally placed on segments I–VIII. Cuticle of body smooth.

***Vestiture***. Setae various in length, hair-like, moderately elongate or short, transparent. Thorax (Fig. 1D): prothorax with 6 *prns* all medium length, equal in size, 2 medium *ps* and 1 minute *eus*. Meso- and metathorax each with 1 short *prs* and 3 various in length *pds*, 1 medium *eps*, 1 medium *ps* and 1 minute *eus*. Pedal areas of thoracic segments each with 3 *pda* of various length (2 medium and 1 short). Abdomen (Fig. 1E, F): segments I–VIII with 2 short *prs*, 3 various in length *pds*, 1 short and 1 medium *ss* (segment VIII with 1 medium *ss* only), 1 medium *eps*, 2 various in length *ps* (1 minute and 1 medium), 1 minute *lsts* and 2 minute *eus*. Abdominal segment IX with 2 medium, equal in length *ds*, 1 minute *ps* and 2 minute *sts* (Fig. 1F). Each lateral lobe of abdominal segment X with single, minute *ts*.

**Figure 2.**
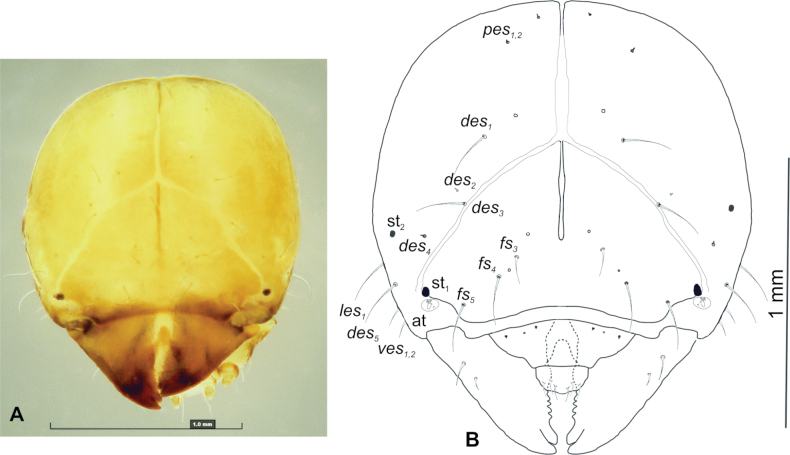
*Tychius amplicollis* mature larva. **A**. Head, dorsal view (photo); **B**. Head, dorsal view (drawing) (*des* – dorsal epicranial s., *fs* – frontal epicranial s., *les* – lateral epicranial s., *pes* – postepicranial s., *ves* – ventroepicranial s., at – antenna, st – stemmata).

***Head capsule*** (Fig. 2A, B) slightly bilaterally narrowed; endocarina elongate, reaches 1/2 of frons; frontal sutures distinct along entire length up to antennae; 2 stemmata (st) visible, first placed at the end of frontal suture, second lateromedially. Setae of head medium to short, hair-like, transparent. Cranial setae: medium *des*_1_ placed medially, *des*_2_ minute placed close to *des*_3_, medium *des*_3_ placed at lateral margin of frontal suture, *des*_4_ minute, medium *des*_5_ placed anterolaterally; *fs*_1_ and *fs*_2_ absent, short *fs*_3_ placed anteromedially, medium *fs*_4_ placed anteromedially, and medium *fs*_5_ placed anterolaterally; medium *les*_1_ placed postero-laterally, *les*_2_ absent, and 2 short *ves*, postepicranial area with 2 minute *pes*.

**Figure 3.**
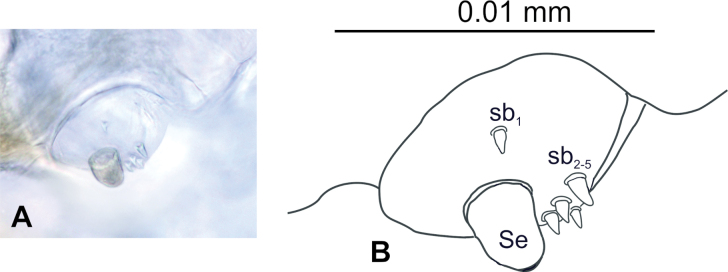
*Tychius amplicollis* mature larva, antenna. **A**. Photo; **B**. Drawing (Se – sensorium, sb – sensillum basiconicum).

***Antennae*** (Fig. 3A, B) placed on each side at anterior margin of head, close to internal border of frontal suture; membranous basal segment convex, semispherical, bearing conical, short sensorium and 5 sensilla basiconica (sb), 1 in middle and 4 on anterior side of basal segment.

**Figure 4.**
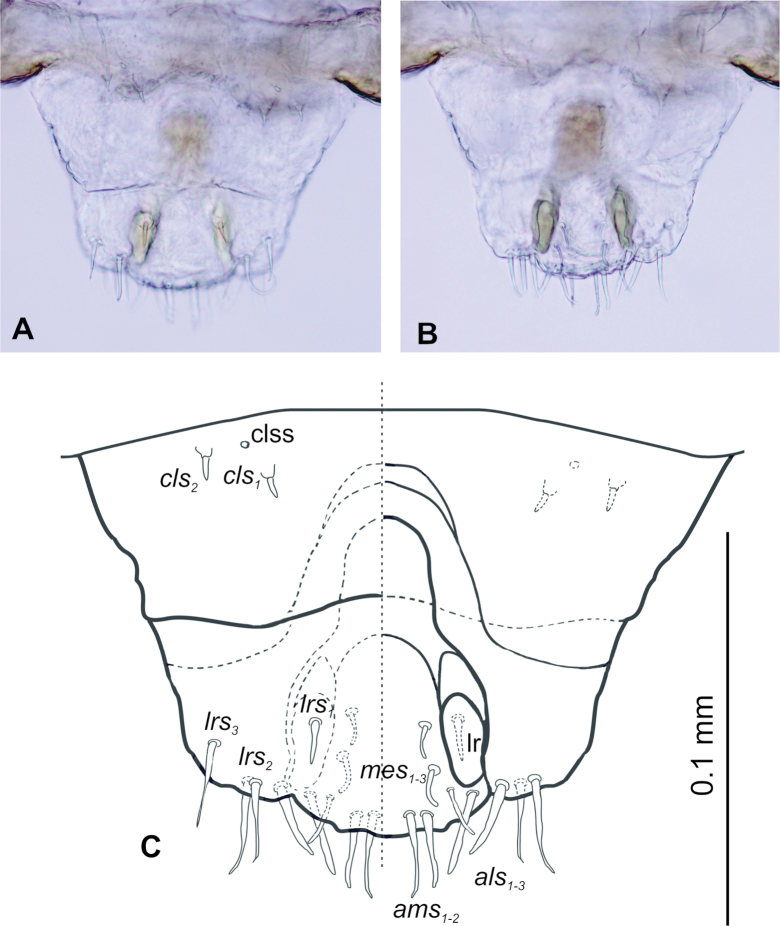
*Tychius amplicollis* mature larva, clypeus and labrum. **A, C**. Left side, clypeus and labrum; **B, C**. Right side, epipharynx (*als* – anteriolateral s., *ams* – anteromedial s., *cls* – clypeal s., *lrs* – labral s., *mes* – median s., lr – labral rods, clss – clypeal sensorium).

***Clypeus*** (Fig. 4A, B left side) trapezoidal, approximately 3.5 times longer than wide, with 2 very short, medially placed *cls* and single sensillum (clss) placed anteriorly between *cls*. Anterior margin of clypeus slightly sinuate.

**Figure 5.**
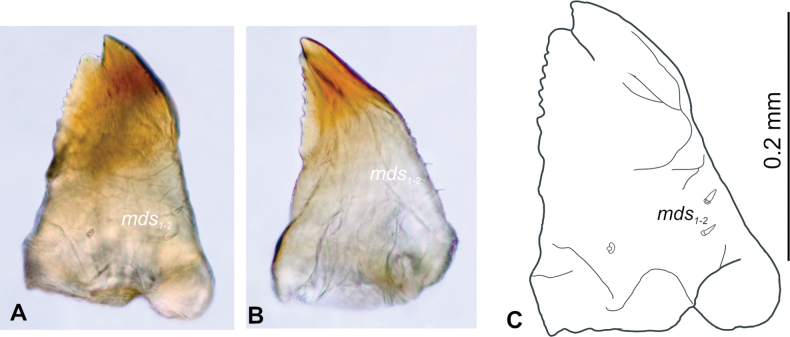
*Tychius amplicollis* mature larva, left mandible. **A**. Dorsal view (photo); **B**. Lateral view (photo); **C**. Dorsal view (drawing) (*mds* – mandible dorsal s.).

**Figure 6.**
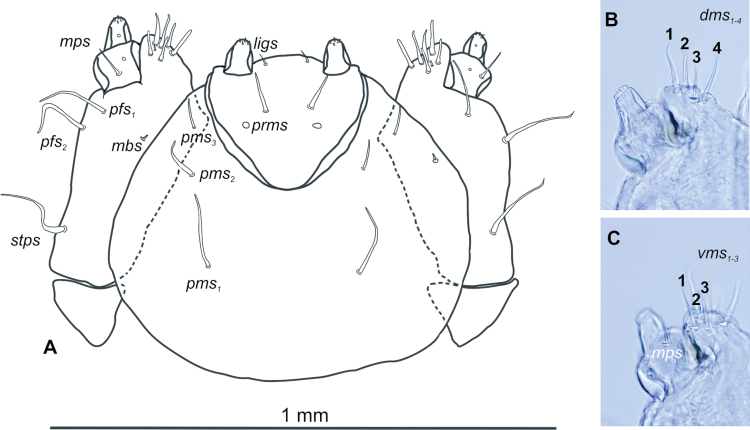
*Tychius amplicollis* larval mouthparts, maxillolabial complex. **A**. Ventral view; **B**. Dorsal view of apical part of maxilla; **C**. Ventral view of apical part of maxilla (*dms* – dorsal malar s., *ligs* – ligular s., *mbs* – basioventral s., *mps* – maxillary palps s., *pfs* – palpiferal s., *pms* – postmental s., *prms* – premental s., *stps* – stipital s., *vms* – ventral malar s.).

***Mouthparts***. Labrum (Fig. 4A, B left side) approximately 2 times longer than wide, anterior margin distinctly sinuate; *lrs*_1_ rather short, medially placed; both *lrs*_2_ and *lrs*_3_ medium, anterolaterally placed. Epipharynx (Fig. 4A, B right side) with 3 *als* and 2 *ams*, all elongate, 3 medium, slightly curved *mes*, equal in size. Labral rods (lr) nearly kidney-shaped, slightly posteriorly converging. Mandible (Fig. 5A–C) rather narrow, bifid, apical tooth much higher than internal one. Cutting edge strongly serrated, without additional protuberance. Setae: *mds*_1_ and *mds*_2_ minute, hair-like, both lateromedially placed. Maxillolabial complex (Fig. 6A–C) moderately wide, on stipes with 1 elongate *stps*, and 2 elongate *pfs*. Mala with 4 *dms*, various in size (1^st^, 2^nd^ and 4^th^ elongate, 3^rd^ short) (Fig. 6B) and a group of 3 various in size *vms* (1^st^ and 2^nd^ sharp, medium, 3^rd^ digitate, small), and 1 minute malar basiventral sensillum (*mbs*) (Fig. 6C). Maxillary palpi with 2 palpomeres; basal palpomere distinctly wider and 1.5 times longer than distal one. Basal palpomere with 1 short *mps* and 1 pore. Distal palpomere with a group of 4 apical sensilla on terminal receptive area (tra). Surface of mala smooth. Labium with subtriangular prementum, with 1 medium prms placed medially. Ligula concave, with 1 minute *ligs*. Premental sclerite nearly ring shaped; postmentum wide, membranous with 3 *pms*: elongate *pms*_1_ situated postero-medially, medium *pms*_2_ placed laterally and short *pms*_3_ placed antero-laterally. Labial palpi 1-segmented. Each palpus with single pore, and a group of 4 apical sensilla on terminal receptive area. Surface of labium smooth.

#### Description of pupa.

***Measurements*** (in mm): body length: 4.20–3.60 (mean 2.60); body width: 2.20–3.00 (mean 2.60); thorax width: 1.30–2.00 (mean 1.50); rostrum length: 1.00–1.50 (mean 1.30).

**Figure 7.**
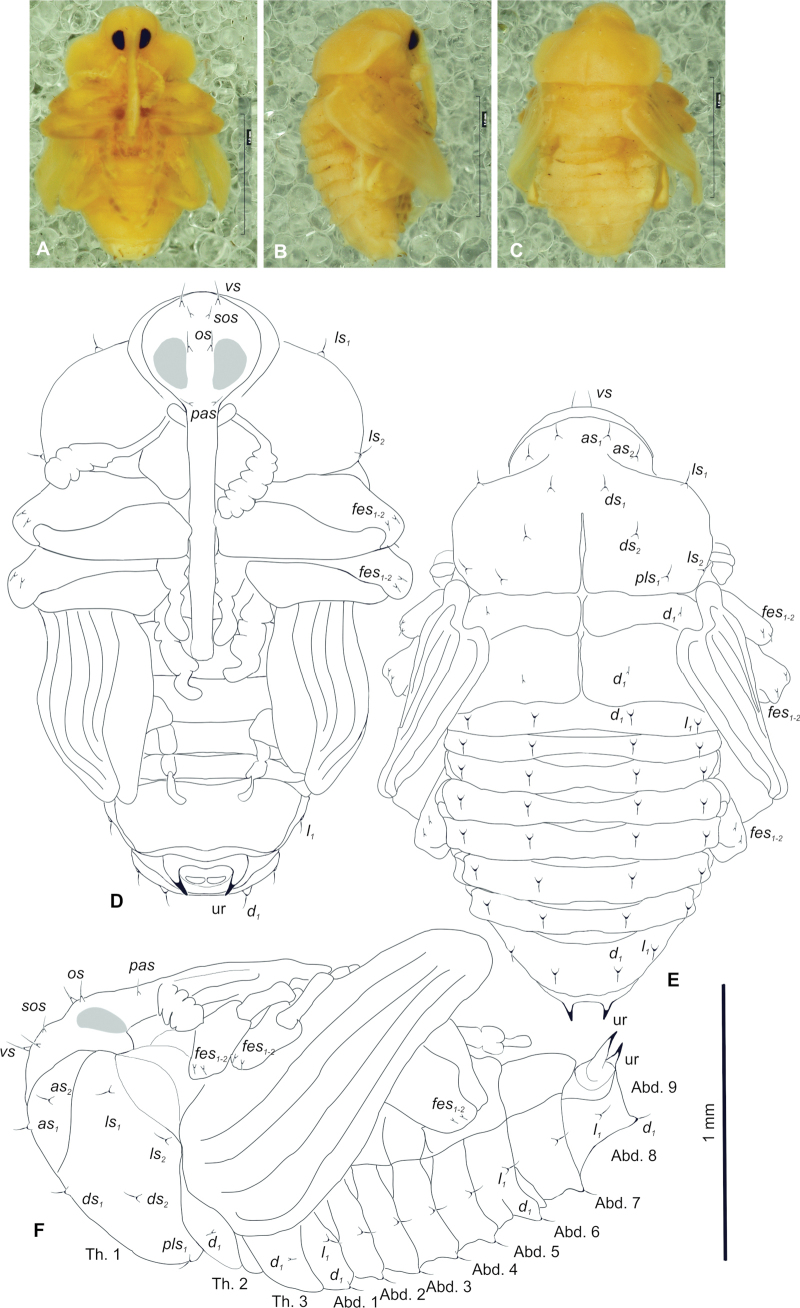
*Tychius amplicollis*, pupa habitus. **A**. Ventral view; **B**. Lateral view; **C**. Dorsal view; **D**. Ventral view; **E**. Dorsal view; **F**. Lateral view (setae; *as* – apical s., *d* – dorsal, *ds* – discal s., *l* (abdomen), *ls* (pronotum) – lateral s., *fes* – femoral; *os* – orbital s., *pls* – posterolateral s., *pas* – postantennal s., *sos* – superorbital s., *vs* – vertical s.; ur – urogomphi, Th.1–3 – number of thoracic segments, Abd.1–10 – number of abdominal seg.). Scale bars: 1 mm.

***Colouration and morphology***. Body yellow, rather stout; cuticle smooth (Fig. 7A–C). Rostrum very elongate, 7.5 times as long as wide in both ♂ and ♀, exceeding metacoxae. Pronotum 1.5 times wider than long, rounded laterally, strongly narrowed apically (Fig. 7A–C). Mesonotum half as long as metanotum. Abdominal segments I–V of equal length, segments VI and VII tapering gradually, segment VIII semicircular, segment VIII narrow, segment IX terminal, with urogomphi (ur) laterally situated, slightly recurved, short, each with sclerotized, sharp apex (Fig. 7A, B). Spiracles placed dorso-laterally on abdominal segments I–VI, functional on segments I–V, vestigial on segment VI.

***Chaetotaxy*** (setal numbers given for 1 side of the body): setae variable in size, hair-like. Rostrum with 1 medium *pas*. Head with 1 medium *os*, 1 short *sos* and 1 medium *vs* (Fig. 7D, F). Pronotum with 2 *as*, 2 *ls*, 2 *ds* and 1 *pls*. All pronotal setae medium. Meso- and metathorax with 1 short seta (*d*_1_) placed medially on dorsum (Fig. 7E, F). Abdominal segments I–VIII each, with 1 medium seta (*d*_1_) placed anteromedially (Fig. 7E, F). All abdominal setae placed on protuberances. Segment IX and urogomphi without setae. Lateral parts of abdominal segments I–VIII with 1 medium seta (*l*_1_) placed medio-laterally single minute setae. Ventral parts of abdominal segments without setae. Each femur with 2 short, hair-like setae (*fes*).

##### Biological observations

The species was collected on *Vicia macrocarpa* (Fabaceae), where the females deposit their eggs within the pods. Larval development occurs inside the seeds. When fully grown, the larvae create a small exit hole in the pod and drop in the ground where they construct a pupal cell using sand grains. Pupation was observed approximately two weeks after the introduction of the larvae into the soil.

*Vicia macrocarpa* is a Mediterranean–Turanian member of the Fabaceae (Kupicha 1981), historically cultivated as forage and consequently considered adventive in Italy, where it is sporadically distributed in all peninsular regions as well as in Piedmont and Liguria (Pignatti 1982; Nimis et al. 2018).

##### Phylogenetic reconstruction

**Figure 8.**
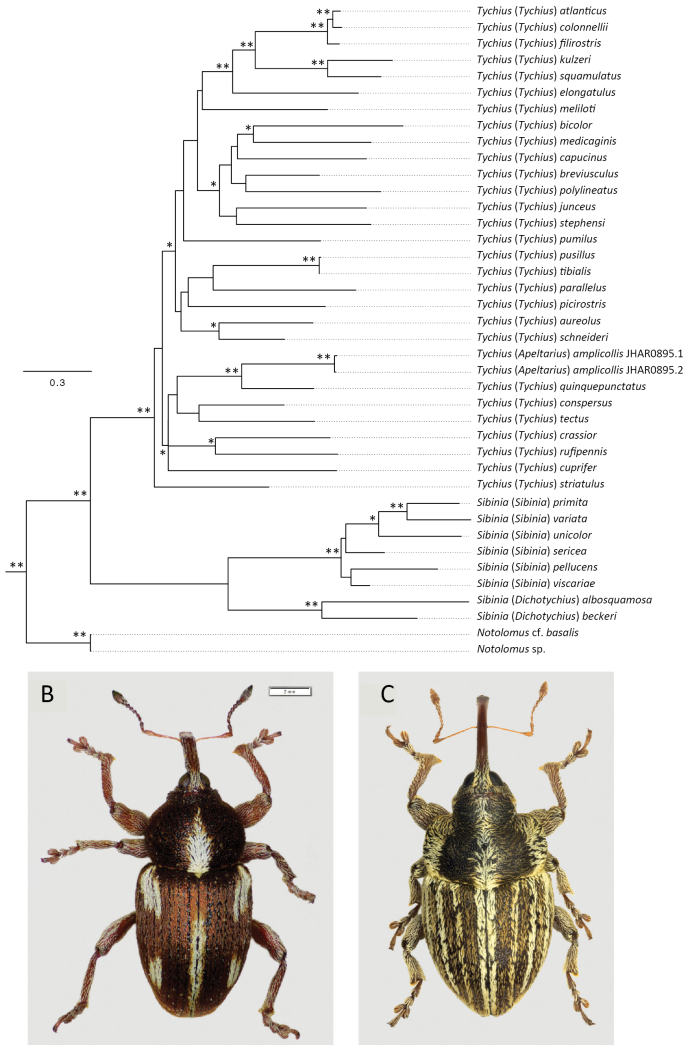
**A**. Maximum-likelihood tree resulting from the analysis of COI Barcode sequences. Support at node refers to SH-aLRT values ≥80 and uBV ≥95% (**). A single asterisk * refers to SH-aLRT values ≥80 only. *Notolomus* specimens are used as an outgroup. Branches crossed out with two slashes ‘//’ have been shortened to improve the clarity of the figure; **B**. *Tychius quinquepunctatus*; **C**. *T. amplicollis*.

Maximum-likelihood analyses based on the COI fragment provided strong support for the monophyly of the genera *Sibinia* and *Tychius* as sampled in this study (Fig. 8A). *Tychius amplicollis* and *Tychius quinquepunctatus* (Fig. 8A, B) were recovered as sister species with strong support and nested well within the genus *Tychius*, but with weak support. Most deeper relationships within this genus were poorly supported.

Just a few years after the description of *Apeltarius* based on a single taxon – *A. multilineatus* (currently *Tychius*) – *Tychius amplicollis* was placed in the same genus by Bedel (1885), due to the very strict similarities of the adults of these two species differing from each other mainly in the shape of the pronotum, which is more strongly constricted at its apex in *T. amplicollis* (Caldara 1978). Whereas *T. multilineatus* is known only from Algeria and doubtfully from Sicily (Baviera and Caldara 2020), *T. amplicollis* has a larger distribution being known from the central part of North Africa, Italy (mainly Sicily and Sardinia, and rare in a few central and southern regions), and doubtfully from Corsica (Caldara 1978; Alonso-Zarazaga et al. 2023).

## Discussion

### Comparison with the immature stages of known Tychiini

Both the larva and pupa of *Tychius amplicollis* exhibit all diagnostic morphological features characteristic of the tribe Tychiini (Skuhrovec et al. 2014, 2015; Gosik et al. 2017; Báborská et al. 2019).

**Larva**. The larva presents the following combination of characters diagnostic of Tychiini: (1) body chaetotaxy strongly reduced; (2) abdominal segments I–VII dorsally divided into three lobes; (3) thoracic spiracle bicameral, abdominal spiracles unicameral; (4) endocarina reaching approximately half the length of the frons; (5) *des*_2_ and *des*_4_ short or absent; (6) *des*_3_ located contiguous to the frontal suture; (7) *fs*_3_ short, *fs*_4_ and *fs*_5_ elongate; (8) epipharynx with 3 *mes*; (9) cutting edge of mandible distinctly jagged; (10) labial palps one-segmented; and (11) premental sclerite ring-shaped or nearly triangular.

**Pupa**. The pupal morphology is likewise consistent with Tychiini, characterized by: (1) strongly reduced chaetotaxy with short or very short setae; (2) pronotum bearing 2 *as*, 2 *ls*, 2 *ds*, and 1 *pls*; (3) abdominal segments I–VIII each with 1 dorsal and 1 lateral seta; (4) abdominal segment IX lacking setae; and (5) urogomphi short.

Within the tribe Tychiini, the larval features that are diagnostic for the genus *Tychius* and are also present in *T. amplicollis*, but absent in other genera such as *Sibinia* Germar, 1817 and *Lignyodes* Dejean, 1835, include: (1) elongate *des*_1_ (vs short or absent in *Sibinia* and *Lignyodes*); (2) epipharynx with 3 *mes* and 2 *als* (vs 2 *mes* and 3 *als* in *Sibinia* and *Lignyodes*); (3) one-segmented labial palps (vs two-segmented in *Lignyodes*); (4) labium with 3 *pms* (vs 2 in *Sibinia*); (5) ring-shaped premental sclerite (vs tridental in *Lignyodes*); (6) meso- and metathorax usually with 1 *ps* (vs 2 in *Sibinia*); (7) head with 1 *les* (vs 2 in *Lignyodes*); and (8) frons with elongate *fs*_5_ (vs short in *Lignyodes*).

Similarly, the pupal characters separating *Tychius* from other Tychiini are as follows: (1) 1 *sos* (vs 2 in *Sibinia*); (2) pronotum with 1 *pls* (vs 2 in *Sibinia* and 3 in *Lignyodes*); (3) abdominal segment IX without setae (vs 1 dorsal seta in *Sibinia* and 2 in *Lignyodes*); (4) pronotal and abdominal setae short or medium in length (vs. elongate in *Lignyodes*); and (5) abdominal segments I–VIII each with 1 dorsal seta (vs 3 dorsally in *Lignyodes*).

These morphological characters constitute clear evidence that *T. amplicollis* fully conforms to the diagnostic concept of the genus *Tychius* and do not justify its placement in a separate genus or subgenus within Tychiini.

### Updated key to the immature stages of *Tychius*

Based on these new observations, the diagnostic key to the immature stages of *Tychius* by Skuhrovec et al. (2014) can be modified as follows:

**Table:** 

**Larva**
8	Abdominal segments I–VII with 2 *prs*	**9**
–	Abdominal segments I–VII with 1 *prs*	**10**
9	Pronotum with 6 *prn*. Meso- and metathorax without *as* and *ss*	**9A**
9A	Abd. segment VIII with 1 *ps*, Abd. segment IX without *ps*, head with 1 *ves* and 1 st; antenna with 2 sensilla, *prms* 1 elongate	***T. quinquepunctatus***
–	Abd. segment VIII with 2 *ps*, Abd. segment IX with 1 *ps*, head with 2 *ves* and 2 st; antenna with 5 sensilla, *prms* 1 short	***T. amplicollis***
**Pupa**
10	Femora with 2 *fes*	**11**
–	Femora with 1 *fes*	**12**
11	Head with 2 *vs* and without *sos*	***T. argentatus***
–	Head with 1 *vs* and 1 *sos*	**11A**
11A	Rostrum with 1 *rs*	***T. quinquepunctatus***
–	Rostrum without *rs*	***T. amplicollis***

### Taxonomic implications and systematic placement

The combination of larval and pupal morphological characters observed in *Tychius amplicollis* (particularly those shared with *T. quinquepunctatus*, the type species of the genus *Tychius*), clearly supports its inclusion within *Tychius*.

Biological observations further reinforce this conclusion: *T. amplicollis* exhibits life habits and host plant associations fully consistent with those of *Tychius*, particularly species feeding on Fabaceae of the tribe Fabeae (Skuhrovec et al. 2014).

Molecular analyses of the COI gene fragment also corroborate these interpretations, as *T. amplicollis* shows a derived phylogenetic position within a clade composed of species belonging to the genus *Tychius*. Given the close morphological relatedness of the four species of the subgenus *Apeltarius*, this finding suggests that this group as a whole (i.e. a species group) is best placed within the genus *Tychius*. Both the genus *Tychius* (including *T. amplicollis*) and its sister genus *Sibinia* were recovered as monophyletic groups with strong support. The internal nodes within *Tychius* show variable support, with several interspecific relationships being weakly supported, but it is worth to note that the close relationship between *T. amplicollis* and *T. quinquepunctatus* is well supported. Further comprehensive studies with expanded taxon sampling and additional genetic markers will be important to further clarify relationships among species of *Tychius*.

### Taxonomic changes

Given this strong morphological, molecular, and ecological congruence, and taking into account that the adult of T. (Apeltarius) amplicollis is without any doubt morphologically very similar to the type species of the subgenus *Apeltarius*, T. (Apeltarius) multilineatus (Caldara 1978), *Apeltarius* should no longer be recognized as a valid subgenus, but treated as a new junior subjective synonym of the genus *Tychius*. Therefore, we propose the following taxonomic changes:

### New synonymy

**Genus *Tychius* Germar, 1817**

= *Apeltarius* Reitter, 1916, syn. nov.

Consequently, the following extant combinations must be recovered:

*Tychius amplicollis* Aubé, 1850.

*Tychius multilineatus* (Desbrochers des Loges, 1873) (formerly *Apeltarius*).

*Tychius quinquelineatus* Tournier, 1874.

*Tychius strigulatus* (Desbrochers des Loges, 1875) (formerly *Apeltarius*).

### Infrageneric placement within *Tychius*

Morphological and biological evidence indicates that the taxa formerly included in the subgenus *Apeltarius* form a coherent homogeneous group within *Tychius*, most closely related to the *T. quinquepunctatus* species group.

In the absence of a comprehensive phylogenetic study for the genus *Tychius*, current intrageneric divisions are based primarily on morphological affinities, which often reflect putative recognized apomorphies (Caldara 1990). Acknowledging the partly subjective nature of such infrageneric classifications, where names of genera and subgenera are governed by the ICZN (1999), but species groups remain informal and pragmatic, the most consistent criterion remains morphological uniformity supported by biological coherence.

Following the grouping principles proposed by Caldara (1990), and in light of the new morphological and molecular evidence presented here, we consider the former *Apeltarius* taxa to represent a distinct species group of *Tychius*. Consequently, these species are here treated as the *Tychius amplicollis* species group, which is closely allied to the *T. quinquepunctatus* species group within the genus *Tychius*.

## Conclusions

In this study, we reassessed the systematic position of the subgenus *Apeltarius* in the genus *Tychius* based on newly obtained information on immature stages, phylogenetic analyses of the COI sequences, and biological observations. The morphological characteristics of the larva and pupa of *T. amplicollis*, together with its bionomic traits, correspond closely to those observed in species of the genus *Tychius*, providing clear evidence of their close relationship.

This work highlights the importance of integrative taxonomy in resolving long-standing systematic issues in entomology. By combining classical morphological studies of adults with detailed analyses of immature stages and supporting molecular and biological data, we achieved a comprehensive understanding of the taxonomic status of the subgenus *Apeltarius*. The synthesis of these complementary lines of evidence strongly supports the conclusion that *Apeltarius* should be regarded as a junior subjective synonym of *Tychius*.

## Supplementary Material

XML Treatment for Tychius amplicollis

## Additional information

### Use of AI

We used AI to correct spelling and grammar mistakes in English.

### Author ORCIDs

Roberto Caldara https://orcid.org/0000-0001-9932-7078

Jiří Skuhrovec https://orcid.org/0000-0002-7691-5990

Gael J. Kergoat https://orcid.org/0000-0002-8284-6215

Roland Godon https://orcid.org/0009-0005-3925-3827

Julien Haran https://orcid.org/0000-0001-9458-3785

Rafał Gosik https://orcid.org/0000-0002-2083-4905

### Data availability

All of the data that support the findings of this study are available in the main text or Supplementary Information.

## Supplementary materials

Supplementary material 1 List of specimens and corresponding accession numbers of COI sequences Data type csv https://binary.pensoft.net/file/1557737This dataset is made available under the Open Database License (http://opendatacommons.org/licenses/odbl/1.0/). The Open Database License (ODbL) is a license agreement intended to allow users to freely share, modify, and use this Dataset while maintaining this same freedom for others, provided that the original source and author(s) are credited. Roberto Caldara, Jiří Skuhrovec, Roberto Casalini, Gael J. Kergoat, Roland Godon, Julien Haran, Rafał Gosik

Supplementary material 2 Multiple sequence alignment of COI gene sequences used for specimen-level analyses Data type fasta https://binary.pensoft.net/file/1557738This dataset is made available under the Open Database License (http://opendatacommons.org/licenses/odbl/1.0/). The Open Database License (ODbL) is a license agreement intended to allow users to freely share, modify, and use this Dataset while maintaining this same freedom for others, provided that the original source and author(s) are credited. Roberto Caldara, Jiří Skuhrovec, Roberto Casalini, Gael J. Kergoat, Roland Godon, Julien Haran, Rafał Gosik

Supplementary material 3 Multiple sequence alignment of COI gene sequences used for species-level analyses Data type fasta https://binary.pensoft.net/file/1557739This dataset is made available under the Open Database License (http://opendatacommons.org/licenses/odbl/1.0/). The Open Database License (ODbL) is a license agreement intended to allow users to freely share, modify, and use this Dataset while maintaining this same freedom for others, provided that the original source and author(s) are credited. Roberto Caldara, Jiří Skuhrovec, Roberto Casalini, Gael J. Kergoat, Roland Godon, Julien Haran, Rafał Gosik

Supplementary material 4 Phylogenetic tree resulting from IQ-TREE analysis of the specimen-level dataset Data type treefile https://binary.pensoft.net/file/1557740This dataset is made available under the Open Database License (http://opendatacommons.org/licenses/odbl/1.0/). The Open Database License (ODbL) is a license agreement intended to allow users to freely share, modify, and use this Dataset while maintaining this same freedom for others, provided that the original source and author(s) are credited. Roberto Caldara, Jiří Skuhrovec, Roberto Casalini, Gael J. Kergoat, Roland Godon, Julien Haran, Rafał Gosik

Supplementary material 5 Phylogenetic tree resulting from IQ-TREE analysis of the species-level dataset Data type treefile https://binary.pensoft.net/file/1557741This dataset is made available under the Open Database License (http://opendatacommons.org/licenses/odbl/1.0/). The Open Database License (ODbL) is a license agreement intended to allow users to freely share, modify, and use this Dataset while maintaining this same freedom for others, provided that the original source and author(s) are credited. Roberto Caldara, Jiří Skuhrovec, Roberto Casalini, Gael J. Kergoat, Roland Godon, Julien Haran, Rafał Gosik
